# IL-22-STAT3-CD155 axis in alveolar echinococcosis: a pivotal role in immune exhaustion and therapeutic potential

**DOI:** 10.3389/fimmu.2025.1674904

**Published:** 2026-01-05

**Authors:** Liang Li, Xue Zhang, Ning Yang, Hui Liu, Junlong Xue, Jin Chu, Guodong Lv, Tuerganaili Aji, Xiaojuan Bi, Renyong Lin

**Affiliations:** 1 State Key Laboratory of Pathogenesis, Prevention and Treatment of High Incidence Diseases in Central Asia, Clinical Medical Research Institute, The First Affiliated Hospital of Xinjiang Medical University, Urumqi, China; 2 Xinjiang Key Laboratory of Echinococcosis, Clinical Medical Research Institute, The First Affiliated Hospital of Xinjiang Medical University, Urumqi, China; 3 Department of Hepatobiliary and Hydatid Diseases, Digestive and Vascular Surgery Center, The First Affiliated Hospital of Xinjiang Medical University, Urumqi, China

**Keywords:** Alveolar echinococcosis, CD155, *Echinococcus multilocularis*, Interleukin-22, macrophage-hepatocyte crosstalk, STAT3 signaling

## Abstract

**Background:**

Alveolar echinococcosis (AE), a lethal zoonosis caused by *Echinococcus multilocularis* (*E.m*) infection, is characterized by immune exhaustion that facilitates parasite evasion of host immunity and sustains chronic infection. The role and mechanisms of Interleukin-22 (IL-22), a key immunomodulatory cytokine, in *E.m*-induced immune responses remain unclear and warrant investigation.

**Methods:**

Liver tissue samples from AE patients and *E.m*-infected mouse models were utilized to investigate IL-22 expression dynamics during AE progression and its correlation with disease progression. Recombinant IL-22 (rIL-22) stimulation and IL-22-binding protein (rIL-22BP) blockade were integrated to comprehensively assess the role of IL-22 through Western blotting, immunohistochemistry (IHC), and enzyme-linked immunosorbent assay (ELISA). Pathological alterations in infected mice were quantified via hematoxylin and eosin (H&E) and Sirius Red staining to evaluate the potential of IL-22 as a therapeutic target. Flow cytometry and *in vitro* co-culture systems were further employed to identify the cellular sources of IL-22 and elucidate its regulatory effects on CD155 expression in hepatocytes.

**Results:**

IL-22 expression was significantly upregulated in liver tissues from both AE patients and *E.m*-infected mice, positively correlating with disease progression. Compared with the infection group (Em), rIL-22 stimulation exacerbated parasitic burden, increasing lesion number and fibrotic area. Conversely, rIL-22BP blockade effectively attenuated pathology, significantly reducing lesion burden and fibrotic area. Mechanistically, *in vitro* co-culture experiments demonstrated that macrophage-derived IL-22 activated STAT3 signaling in hepatocytes, upregulating CD155 expression, which is a key mechanism underlying *E.m*-induced immune exhaustion. rIL-22BP treatment disrupted IL-22-CD155 intercellular crosstalk, promoting CD8^+^ T-cell recruitment to lesions and reversing their exhausted state.

**Conclusion:**

Our study demonstrates that the IL-22–STAT3–CD155 axis, mediated by macrophage-hepatocyte crosstalk, drives the establishment of an immune-exhaustive microenvironment during *E.m* infection. Mechanistically, macrophage-derived IL-22 induces CD155 upregulation in hepatocytes via IL-22RA1/STAT3 signaling. Critically, rIL-22BP blockade disrupts this axis, reversing the immunosuppressive cascade, restoring CD8^+^ T-cell effector functions, and remodeling the immune microenvironment. This intervention ultimately enhances host-mediated clearance of *E.m*.

## Introduction

Alveolar echinococcosis (AE), a zoonotic parasitic disease caused by the larval-stage infection of *Echinococcus multilocularis* (*E.m*), is endemic in temperate and subarctic regions of the Northern Hemisphere and is characterized by invasive hepatic growth and insidious early symptoms (1). Over 90% of patients progress to lethal late-stage lesions upon diagnosis, with a 5-year mortality rate exceeding 90%, leading to its classification by the World Health Organization (WHO) as a Neglected Tropical Disease requiring urgent intervention (2). AE progression is intrinsically linked to host immune status, with the innate immune system, including macrophages and neutrophils, initially suppressing parasite proliferation through reactive oxygen species and pro-inflammatory cytokines such as Interferon-alpha (IFN-α) and Interleukin-6 (IL-6) (3, 4). However, *E.m* secretes immunomodulatory proteins (e.g., antigen B) to polarize Th2-type immunity, thereby promoting anti-inflammatory cytokines (Interleukin–10, IL-10; transforming growth factor-beta, TGF-β) and fostering an immunosuppressive microenvironment that facilitates immune evasion. Disruption of immune homeostasis, a pivotal driver of AE pathogenesis, underscores the necessity to elucidate the dynamic interplay between host immunity and parasitic immune escape mechanisms to provide a rationale for developing targeted immunotherapies.

Interleukin-22 (IL-22), a member of the IL-10 cytokine family produced by T cells, innate lymphoid cells (ILCs) and macrophages, plays context-dependent roles in diverse non-neoplastic liver diseases, exhibiting dual pro- and anti-inflammatory functions across disease models (5–8). Studies have reported that IL-22 is significantly upregulated in patients with liver fibrosis (9–11). IL-22 signals through a heterodimeric receptor complex comprising IL-22 receptor1 and IL-10 receptor2, and is regulated by the endogenous antagonist IL-22-binding protein (rIL-22BP) that inhibits its interaction with IL-22 receptor1 (12–14). During inflammatory responses, IL-22 binding to its receptor complex on kupffer cells (KCs) suppresses their polarization toward pro-inflammatory M1 phenotypes via signal transducer and activator of transcription factor 3 (STAT3) activation (15). Additionally, IL-22 serves as a critical factor for tissue repair, maintaining liver homeostasis through STAT3-dependent pathways (5, 16). Early studies in *Echinococcus granulosus* infection suggest IL-22 may contribute to host defense by activating Th22 responses and enhancing epithelial barrier integrity, potentially cooperating with Th17/AhR pathways in immune homeostasis regulation (17). However, the role of IL-22 in AE remains poorly defined.

Previous studies have demonstrated that Th cell-derived IL-22 induces CD155 overexpression in lung tumor cells, impairing natural killer (NK) cell function via CD226 internalization and promoting tumor metastasis (18). Our prior research revealed a pronounced immunosuppressive microenvironment surrounding hepatic lesions in both AE patients and *E.m*-infected mouse models, driven by the CD155-TIGIT (T cell immunoreceptor with Ig and ITIM domains, TIGIT)axis, which mediates immune evasion (19). Furthermore, TIGIT^+^ NK cells reinforce local immunosuppression activity through surface programmed death ligand 1 expression and IL-10 secretion (20). Nevertheless, it remains unknown whether IL-22 participates in AE-associated immune evasion by modulating the CD155-TIGIT axis or regulating Th2/Treg mediated responses.

## Methods

### Clinical specimens

This study enrolled 54 patients with AE and 75 healthy volunteers. Patient specimens were obtained from individuals admitted to the First Affiliated Hospital of Xinjiang Medical University between 2018 and 2021, who were diagnosed with liver biopsy and underwent surgical resection (excluding those with immunosuppression-related disorders). The 54 AE patients were grouped following the WHO Informal Working Group on Echinococcosis PNM classification (21). Blood and liver tissue samples were collected from AE patients, while only blood samples were obtained from healthy volunteers. Liver tissue specimens were collected during surgery from peri-lesional areas, specifically including liver tissue close to the parasitic lesion (CLT) and distant normal liver tissue (DLT) remote from the lesion site. The study protocol was approved by the hospital’s Ethics Committee (Approval No. 20160114–12), with all participants providing written informed consent in accordance with the World Medical Association Declaration of Helsinki (1975). Baseline characteristics of AE patients are detailed in **Table 1**, **Supplementary Table S1**.

**Table 1:** Baseline characteristics of the patients and health control.

Characters\Groups	Health control (n=75)	HAE (n=54)
Age	42.93 ± 8.60	39.11 ± 13.96
Gender		
Male	37 (49.33%)	28 (51.85%)
Female	38 (50.67%)	26 (48.15%)
WHO PNM stage		
P1		1
P2		10
P3		18
P4		25
N0		35
N1		19
M0		47
M1		7

PNM, P=location of the parasitic mass in the liver, N=involvement of neighboring organs, M=metastases; HAE, Human Alveolar Echinococcosis.

### Experimental animals

Fifty female C57BL/6 mice (7–8 weeks old) were purchased from Beijing Viton Lihua Laboratory Animal Science and Technology Co. and randomly divided into six groups: the sham operation group (Sham), the infection group (Em), the infection-combined recombinant IL-22 protein (rIL-22) stimulation group, the infection-combined recombinant IL-22BP protein(rIL-22BP) intervention group with 10 mice in each group, the macrophage clearance by clodronate liposomes group (Em-CL) and vehicle group (Em-PBS) with 5 mice per group. Animals were housed under specific pathogen-free (SPF) conditions in a constant temperature and humidity environment in the Laboratory Animal Center of Xinjiang Medical University. All procedures strictly adhered to the ARRIVE guidelines 2.0 and were approved by the Animal Ethics Committee of the First Affiliated Hospital of Xinjiang Medical University (K202110-18).

### *In vivo* experimental design and grouping

Protoscoleces (PSCs) were isolated from Mongolian gerbil intraperitoneal lesions as described previously (22). To establish *E.m* infection, mice in the Em, rIL-22, rIL-22BP, Em-CL, and Em-PBS groups received an injection of 2000 PSCs each via the hepatic portal vein, while the Sham group received an equal volume of saline. Two weeks post-infection, mice in the rIL-22 and rIL-22BP groups began weekly intraperitoneal injections of rIL-22 protein (rIL-22, 0.5 mg/kg) or rIL-22-BP (rIL-22BP, 0.5 mg/kg) respectively, and the Em group received saline. The dose of rIL-22 was selected based on its established efficacy in a mouse model of liver injury, as described by Xiang et al. (23). Mice in the Sham Em, rIL-22, and rIL-22BP groups were sacrificed at 1 month and 3 months (5 mice per group) after infection for liver and serum sampling. For macrophage clearance in *E.m*-infected mice, the Em-CL group received intraperitoneal clodronate liposomes (CL, 100 μl/10 g body weight) as described previously (3), followed by PSCs injection via the hepatic portal vein 3 days later, with weekly CL administration continued for 4 weeks to sustain clearance. The Em-PBS group received phosphate buffered saline liposomes (PL) at the same time points. Both Em-CL and Em-PBS groups were sacrificed at 1-month post-infection. The entire liver lesions were excised and weighed, and the number and area of parasitic lesions on the liver surface were measured and recorded to assess parasite load.

### Serum ALT and AST assay

Fresh serum samples collected from mice were centrifuged at 1500 × g for 5 min at 4°C to remove debris. Serum levels of alanine aminotransferase (ALT) and aspartate aminotransferase (AST) were then measured with a standardized enzymatic colorimetric method according to the manufacturer’s protocols (BC1555, Glutamic-pyruvic Transaminase Activity Assay Kit; BC1565, Glutamic-oxalacetic Transaminase Assay Kit, Solarbio life Sciences, Beijing, China).

### Co-culture assay with RAW264.7 and AML12 cells

The murine macrophage cell line RAW264.7 (CL-0190) and the murine hepatocyte cell line AML12 (CL-0602) were purchased from Procell Life Science & Technology Co., Ltd. (Wuhan, China). For co-culture experiments, RAW264.7 cells were divided into co-culture and separate-culture groups; the co-culture group received PSCs at a 1:500 ratio (PSCs: cells) after viability confirmation (> 95% by trypan blue exclusion). This ratio was selected based on preliminary titration experiments and supported by existing literature demonstrating its utility in inducing macrophage polarization (24). After 24 h of co-culture, conditioned medium from both groups was collected. AML12 cells were then assigned to four treatment groups to dissect the source of signals influencing CD155 expression (1): Control (2), PSCs-Sup (supplemented with conditioned medium from PSCs alone) to assess the direct effect of *E.m*-derived factors (3), Mac-Sup (supplemented with conditioned medium from separately cultured RAW264.7 cells) to control for factors from naïve macrophages, and (4) PSCs+Mac-Sup (supplemented with conditioned medium from RAW264.7 cells co-cultured with PSCs) to investigate the indirect effect mediated by *E.m*-educated macrophages. Each conditioned medium was added to AML12 cultures for 48 h, after which cells were harvested for downstream analyses.

### Flow cytometry analysis

Harvested AML12 cells from each group were stained with an anti-mouse CD155-PE/Cy7 antibody (131512, Biolegend, USA). Data acquisition was performed on a BD FACSAria II flow cytometer (BD Immunocytometry Systems, San Diego, CA), and subsequent analyses were conducted using FlowJo software (version 7.6.1, Tree Star, San Carlos, CA).

### Western blotting

AML12 cells from each group were lysed in RIPA buffer supplemented with protease inhibitors (87787, Thermo Fisher, USA). Protein concentrations were determined using the BCA Protein Assay Kit (23225, Thermo Fisher, USA). Equal amounts (10 µg per lane) were resolved on 10% TGX Stain-Free polyacrylamide rapid gel preparation kit (Bio-Rad, USA, 1610183) and electro-transferred onto 0.45 µm PVDF membranes (IPVH00010, Millipore, Germany). After blocking with 5% non-fat milk in TBST (pH 7.6) for 2 h at room temperature, membranes were incubated overnight at 4°C with the following primary antibodies: anti-IL-22RA1 rabbit polyclonal antibody (GB11302-100, 1:1000, Servicebio), anti-STAT3 rabbit polyclonal antibody (GB11176-100, 1:1000, Servicebio), and anti-CD155 rabbit polyclonal antibody (31447, 1:1000, Proteintech). Following secondary antibody incubation and chemiluminescent detection, band intensities were quantified with ImageJ (v1.54, NIH, USA) and normalized to GAPDH. Data were presented as mean ± SD.

### Enzyme-linked immunosorbent assay

IL-22 levels in sera from AE patients, healthy volunteers, mice, and cell-culture supernatants were quantified with commercial enzyme-linked immunosorbent assay (ELISA) kits (BMS2047 and 88-7422-22, Thermo Fisher, USA) according to the manufacturers’ protocols. CD155 levels in sera from AE patients and healthy volunteers were quantified with the Human CD155/PVR ELISA Kit (EH79RB, Thermo Fisher, USA).

### Hematoxylin–Eosin and Sirius Red staining

Liver specimens from AE patients and mice were fixed in 4% paraformaldehyde, dehydrated, cleared, and embedded in paraffin. Serial 4 µm sections were prepared and stained with H&E (G1120, Solarbio, Beijing, China) to evaluate general histopathology, or with modified Sirius Red (G1472, Solarbio, Beijing, China) to quantify collagen deposition and fibrotic area.

Liver fibrosis was quantified by measuring the Sirius Red-positive area on histological sections. For each animal (n=5 per group), at least 3 random fields (200x magnification) were captured from different lobes of the liver. The images were quantitatively analyzed using the Image-Pro Plus software (Version 6.0.0.260, Media Cybernetics, USA). The area of positive Sirius Red staining was determined by applying a consistent color threshold that was manually set to cover all fibrillar collagen deposits in a blinded manner. The same threshold setting was then applied to all images within the same experimental batch. The percentage of fibrotic area (Sirius Red-positive area/total tissue area) was calculated for each field, and the average value for each animal was used for statistical analysis.

### Immunohistochemistry

After deparaffinization and rehydration, 4 µm liver sections were subjected to heat-induced epitope retrieval in citrate buffer (ZLI-9064, ZSGB-BIO, Beijing, China). Sections were blocked for 1 h at room temperature with PBS containing 10% goat serum (ZLI-9056, ZSGB-BIO, Beijing, China), then incubated overnight at 4 °C with the following primary antibodies: anti-IL-22 rabbit pAb (GB11259-100, Servicebio), anti-IL-22RA1 rabbit pAb (GB11302-100, Servicebio), Anti-STAT3 Rabbit pAb (GB11176-100, Servicebio), Anti-p-STAT3 Rabbit pAb (GB150001-100, Servicebio), anti-CD155 rabbit pAb (31447, Proteintech), Anti-TIGIT Rabbit mAb (ab300073, Abcam), Anti-F4/80 Rabbit pAb (29414, Proteintech), and anti-CD8α rabbit mAb (ab217344, Abcam). The next day, sections were incubated for 2 h at room temperature with HRP-conjugated goat anti-rabbit IgG (ab6013, Abcam). Immunoreactivity was visualized with the DAB substrate kit (ab64238, Abcam) according to the manufacturer’s protocol. Three random fields per section were analyzed with the Image-Pro Plus software (Version 6.0.0.260, Media Cybernetics, USA) based on positively stained areas.

### Statistical analysis

All analyses were performed with GraphPad Prism 10 (GraphPad Software, San Diego, CA). Differences between two groups were evaluated by unpaired or paired Student’s t-tests as appropriate, whereas multiple groups were compared using one-way ANOVA followed by Sidak’s multiple-comparison test. Correlations were assessed using Pearson’s correlation analysis. Data are presented as mean ± standard deviation (SD), and *P* < 0.05 was considered statistically significant. (*P*-values were expressed as follows: *P* < 0.05; *P* < 0.01; *P* < 0.001; *P* < 0.0001).

## Results

### IL22 is elevated both in the AE patients and *E.m-*infected mice

**Figure 1:**
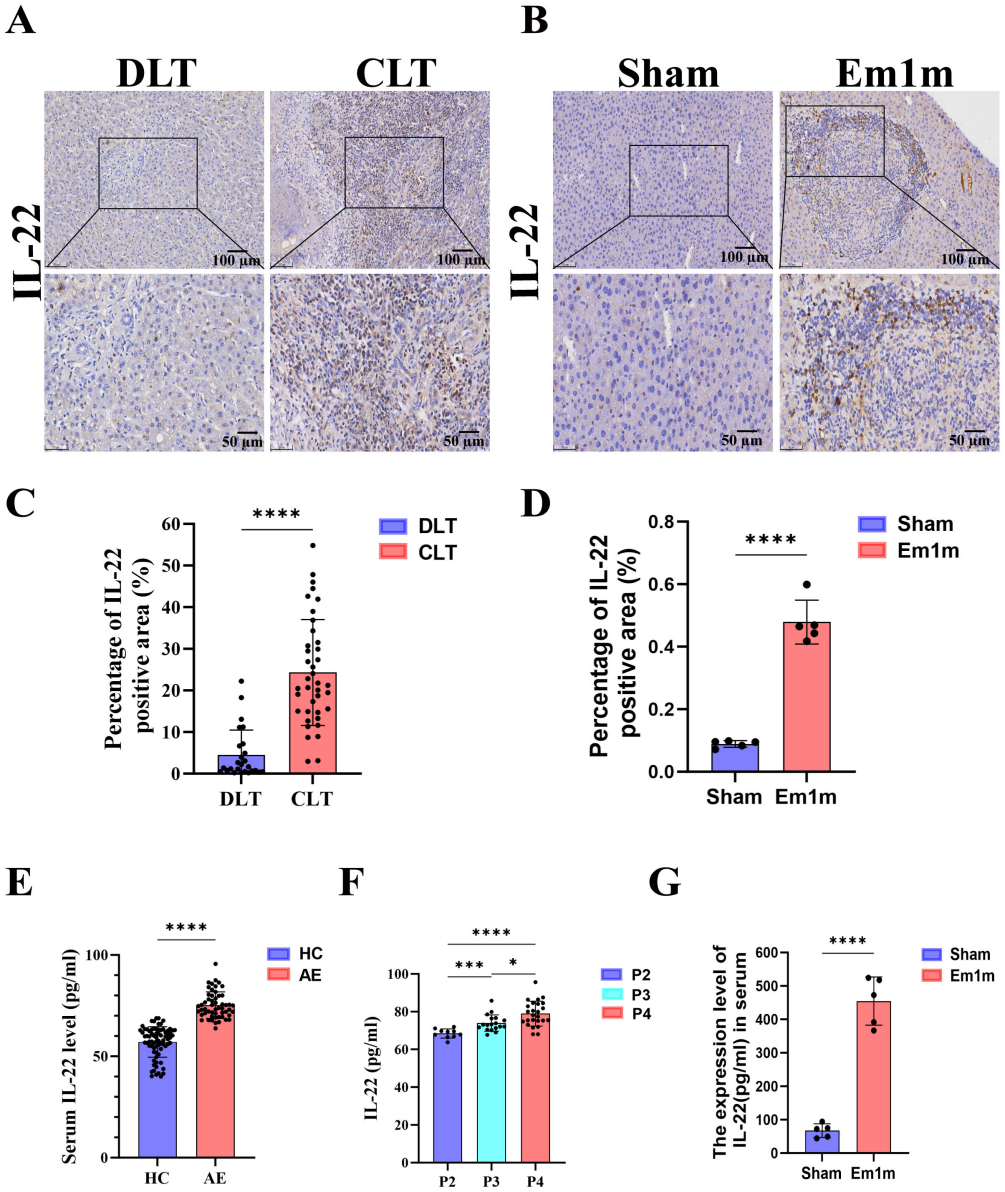
IL-22 is elevated in the liver and serum of alveolar echinococcosis (AE) patients and *Echinococcus multilocularis* (*E.m*) infected mice. **(A)** Immunohistochemical representative images of IL-22 in the adjacent (CLT) and distal liver tissues (DLT) of AE patients. Upper row scale bar: 100μm, lower row scale bar: 50μm. **(B)** Immunohistochemical representative images of IL-22 in the liver tissues of mice at 1-month post-infection. Upper row scale bar: 100μm, lower row scale bar: 50μm. **(C)** Statistical results of the positive area of IL-22 immunohistochemistry in AE patients (n =39). **(D)** Statistical results of the positive area of IL-22 immunohistochemistry in mice at 1-month post-infection (n = 5). **(E)** The expression level of IL-22 in the serum of AE patients and healthy volunteers (n:HC=75 vs AE = 54). **(F)** The expression level of IL-22 in the serum of AE patients at different disease stages (n: P2 = 10 vs P3 = 18 VS P4 = 25). **(G)** The serum expression level of IL-22 in mice at 1-month post-infection (n =5). CLT, Close liver tissues; DLT, Distance liver tissues; Sham, non-infected group; Em1m, Em -infected 1 mouth group; HC, Health control; AE, Alveolar echinococcosis patients; P2, Central lesions with proximal vascular and/or biliar involvement of one lobe; P3, Central lesions with hilar vascular or biliar involvement of both lobes and/or with involvement of two hepatic veins; P4, Any liver lesion with extension along the vesselsb and the biliary tree. *P < 0.05, ***P < 0.001, ****P < 0.0001.

To clarify the role of IL-22 in AE progression, we collected liver tissues from AE patients (**Supplementary Table S1**). Immunohistochemical analysis revealed significantly higher IL-22 expression in CLT than in DLT (**Figures 1A, C**). Moreover, serum IL-22 levels in AE patients were markedly elevated relative to those in healthy volunteers (**Figure 1E**), and these increases were positively correlated with advancing PNM stages (**Figure 1F**). *In vivo* experiments further demonstrated that hepatic and serum IL-22 levels in Em1m group were substantially higher than in Sham group (**Figures 1B, D, G**). Collectively, these findings indicate that IL-22 is actively involved in AE pathogenesis and that its expression correlates positively with disease severity.

### Blockade of IL22 signaling significantly alleviates liver injury in *E.m*-infected mice

**Figure 2:**
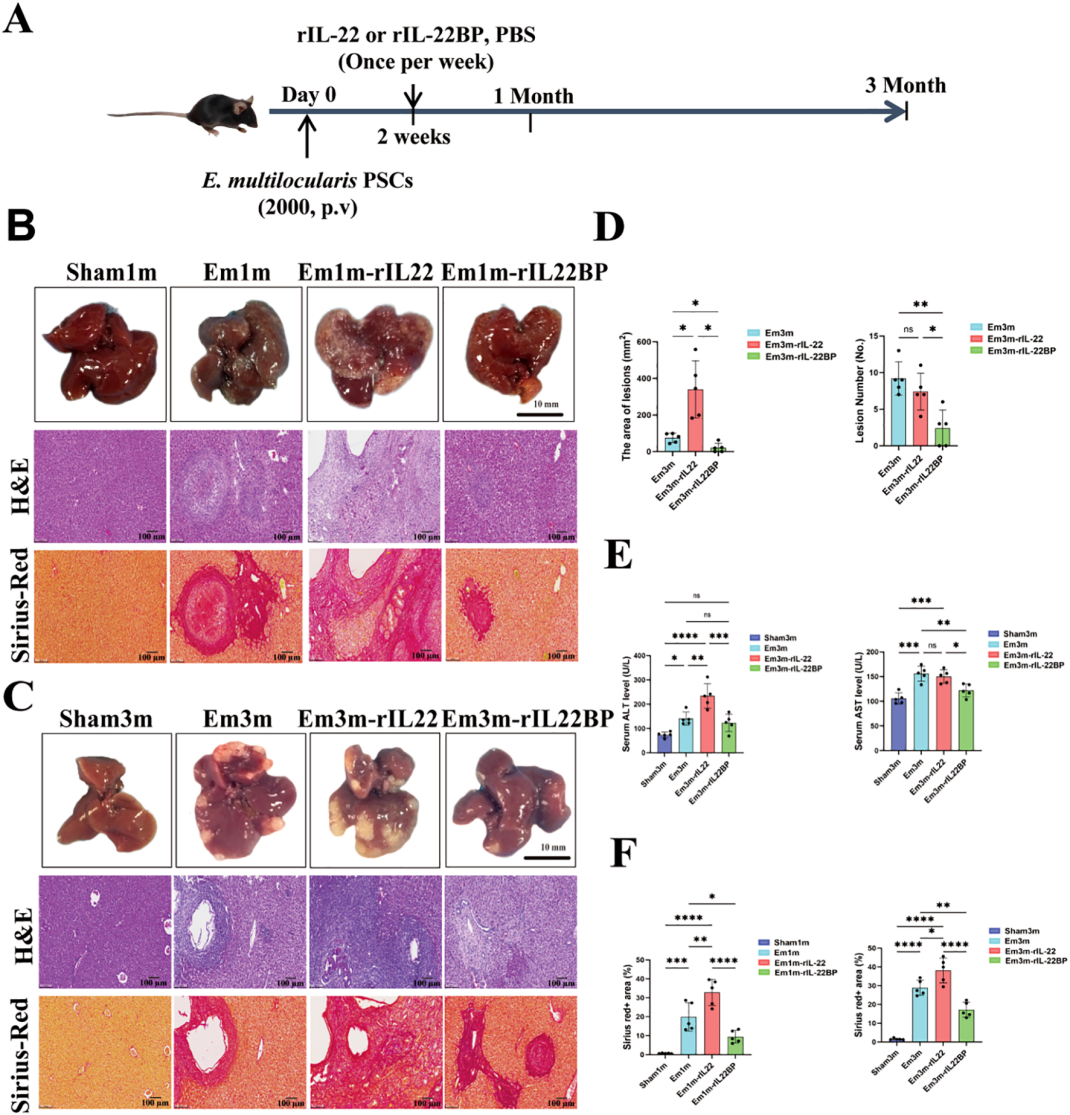
The influence of IL-22 intervention on liver injury in *E.m* infected mice. **(A)** Schematic diagram of the IL-22 intervention experiment in mice. **(B)** Gross images of liver tissues (Scale bar: 10 mm) and representative images of H&E and Sirius red staining (Scale bar: 100μm) in each group of mice at 1-month post-infection. **(C)** Gross images of liver tissues (Scale bar: 100μm) and representative images of H&E and Sirius red staining (Scale bar: 100μm) in each group of mice at 3-month post-infection. **(D)** Statistical results of the area and number of leisons in each group of mice at 3-month post-infection (n =5). **(E)** Statistical results of ALT and AST levels in the serum of mice at 3-month post-infection (n = 5). **(F)** Statistical results of the positive area of Sirius red staining in each group of mice at 1-and 3-month post-infection (n = 5). Sham1m, non-infected 1 mouth and PBS-treated group; Em1m, Em-infected 1 mouth group; Em1m-rIL22, Em-infected 1 mouth and rIL-22 treated group; Em1m-rIL22BP, Em-infected 1 mouth and rIL-22BP treated group; *P < 0.05, **P < 0.01, ***P < 0.001, ****P < 0.0001. ns, no significance.

To elucidate the role of IL-22 in liver injury caused by AE, we generated an *E.m-*infected murine model and manipulated IL-22 bioactivity by administering recombinant IL-22 protein (rIL-22 group) or its high-affinity inhibitor rIL-22BP (rIL-22BP group), thereby interrogating the impact of IL-22-directed intervention on parasite infection (**Figure 2A**). Gross observation revealed conspicuous parasitic lesions on the liver surface of all infected groups at 1-month post-infection lesions appeared as scattered diminutive white nodules with diameters < 1.0 mm, which enlarged to 1.5-3.0 mm and fused at 3 months post-infection (**Figures 2B, C**). Liver weight and hepatic index were persistently elevated in infected groups relative to Sham group and were further augmented in rIL-22 group, yet this escalation was effectively restrained in rIL-22BP group at 3-month post-infection (**Supplementary Figure S1**). Mice in Em3m-rIL22BP group exhibited markedly fewer and smaller surface lesions compared with mice in Em3m and Em3m-rIL22 groups at 3 months post-infection (**Figure 2D**). Sirius red staining demonstrated robustly increased fibrotic areas in infected groups (Em1m and Em3m) versus Sham group. IL-22 supplementation intensified the fibrosis whereas rIL-22BP intervention significantly attenuated collagen deposition with the most pronounced protection observed at 3 months post-infection (**Figure 2F**). Serological tests indicated that AST and ALT levels in Em3m group surpassed those in Sham group and IL-22 administration further elevated ALT whereas rIL-22BP blockade restored both transaminases’ levels close to those in Sham group (**Figure 2E**). These findings collectively demonstrate that IL-22 serves as a pivotal driver of hepatic injury during *E.m* infection, and targeted inhibition of IL-22 simultaneously suppresses parasitic lesion expansion while mitigating liver fibrogenesis.

### Macrophage clearance significantly reduces IL-22 levels in *E.m-infected* mice

**Figure 3:**
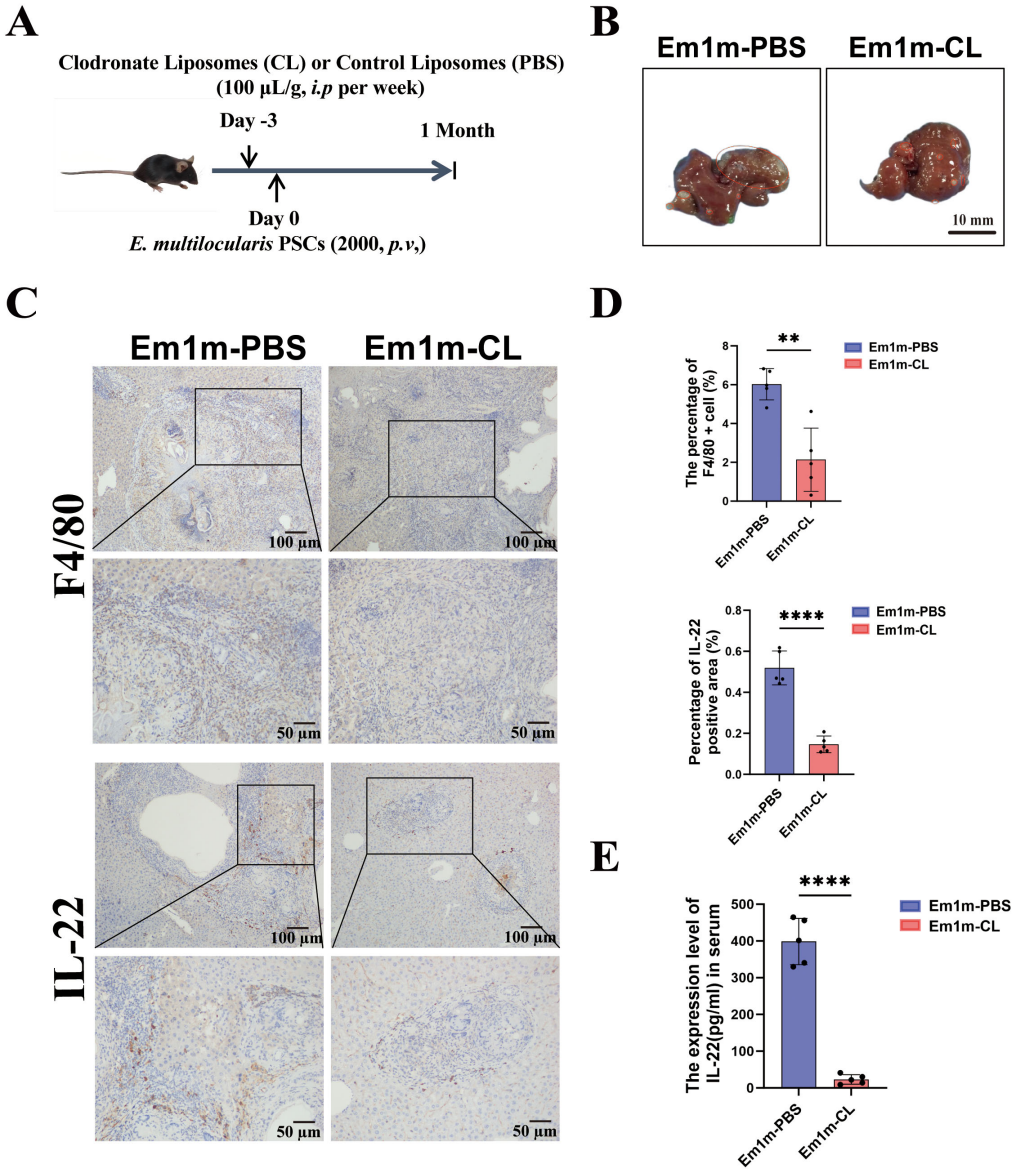
Macrophage clearance reduced the expression of IL-22 in both liver and serum of *E.m* infected mice. **(A)** Schematic diagram of the macrophage clearance experiment in *E.m* infected mice. **(B)** Gross images of liver tissues in mice at 1-month post-infection. Scale bar: 10 mm. **(C)** Immunohistochemical representative images of F4/80 and IL-22 in the liver tissues of mice at 1-month post-infection. Upper row scale bar: 100μm, lower row scale bar: 50μm. **(D)** Statistical results of the proportion of F4/80^+^ and IL-22^+^ cells in the liver of mice at 1-month post-infection (n =5). **(E)** The serum expression level of IL-22 in mice at 1-month post-infection (n =5). Em1m-PBS, Em-infected 1 mouth and PBS treated group; Em1m-CL, Em-infected 1 mouth and Clodronate liposomes treated group; **P < 0.01, ****P < 0.0001.

To identify the cellular source of IL-22 in *E.m-*infected mice, we established a macrophage-depleted infected mouse model using clodronate liposomes (CL). Mice treated with PBS liposomes served as the control group (**Figures 3A, B**). Immunohistochemical revealed that hepatic F4/80^+^ macrophage infiltration was markedly reduced in the Em-CL group, accompanied by a concomitant decrease in the IL-22^+^ area (**Figures 3C, D**). Consistently, serum IL-22 concentrations were significantly lower in the Em-CL group compared with the Em-PBS group (**Figure 3E**). Collectively, these data indicate that macrophages are the major cellular source of IL-22 during *E.m* infection.

### PSCs-induced macrophage derived conditioned medium promotes CD155 expression in hepatocytes

**Figure 4:**
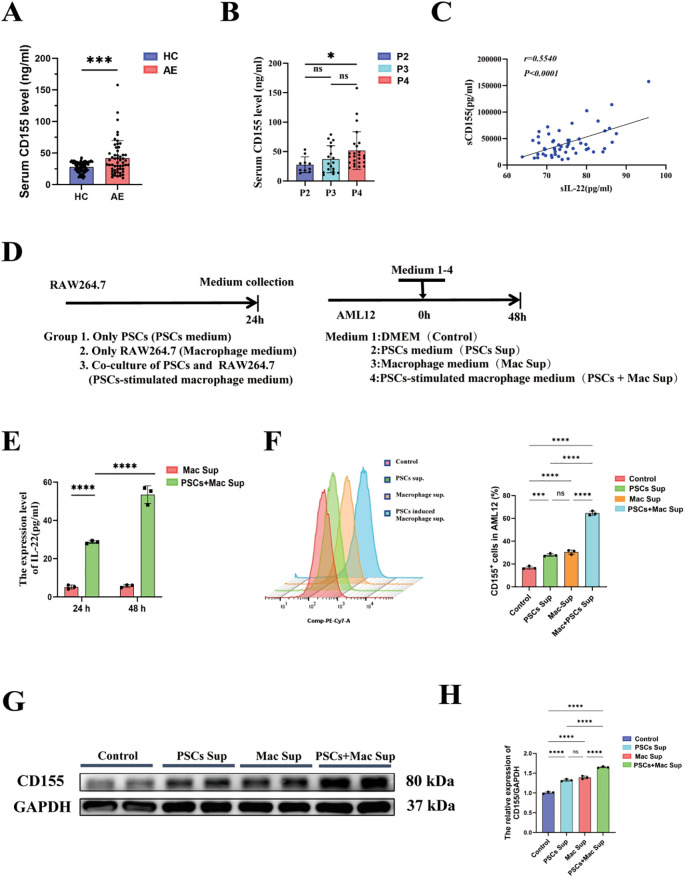
Supernatant from PSCs-stimulated macrophages significantly upregulated CD155 expression in hepatocytes. **(A)** The expression level of CD155 in the serum of AE patients and healthy volunteers (n: HC = 75 vs AE = 54). **(B)** The expression level of CD155 in the serum of AE patients at different disease stages (n: P2 = 10 vs P3 = 18 VS P4 = 25). **(C)** Correlation analysis results of CD155 and IL-22 expression in serum (n=54). **(D)** Schematic diagram of the macrophage-hepatocyte intervention experiment. **(E)** Quantitative results of ELISA to detect IL-22 expression levels in conditioned medium (n = 3). **(F)** Quantitative results of CD155^+^ hepatocyte proportions detected by flow cytometry across experimental groups (n =3). **(G)** Representative western blotting images of CD155 expression in hepatocytes across experimental groups. **(H)** Statistical results of CD155 expression in hepatocytes across experimental groups (n =3). *P < 0.05, ***P < 0.001, ****P < 0.0001.

Serum from AE patients contained significantly higher soluble CD155 (sCD155) compared to healthy volunteers (**Figure 4A**), with these levels closely correlating with PNM clinical stages (**Figure 4B**). Consistent with findings in breast and lung cancer, by Briukhovetska et al. (18), sCD155 correlated strongly with circulating IL-22 (**Figure 4C**), pointing to IL-22 as a potential driver of CD155 expression. To dissect the macrophage-hepatocyte crosstalk that underpins this regulation, AML12 hepatocytes were exposed to distinct conditioned medium (**Figure 4D**). ELISA revealed that supernatants from PSCs-stimulated RAW264.7 macrophages (PSCs+Mac Sup group) contained markedly more IL-22 than those from unstimulated macrophages (Mac Sup group) (**Figure 4E**). After being treated with different groups, the absence of significant cytotoxicity across treatments confirmed that the observed effects were specific biological responses. Flow cytometry showed that PSCs Sup, Mac Sup and, most prominently, PSCs+Mac Sup elevated the proportion of CD155^+^ hepatocytes compared with Control group (**Figure 4F**). Western blotting analysis further confirmed that PSCs+Mac Sup treatment inducing the highest CD155 protein abundance among all conditions (**Figures 4G, H**). Collectively, these results indicate that macrophages, particularly those stimulated by PSCs, secrete IL-22 to directly induce CD155 expression in hepatocytes. This suggests that IL-22-CD155 intercellular crosstalk serves as a pivotal regulatory mechanism in establishing host immune tolerance.

### IL-22-IL-22RA1-STAT3 signaling axis is involved in the high expression of CD155 in *E.m-*infected mice

**Figure 5:**
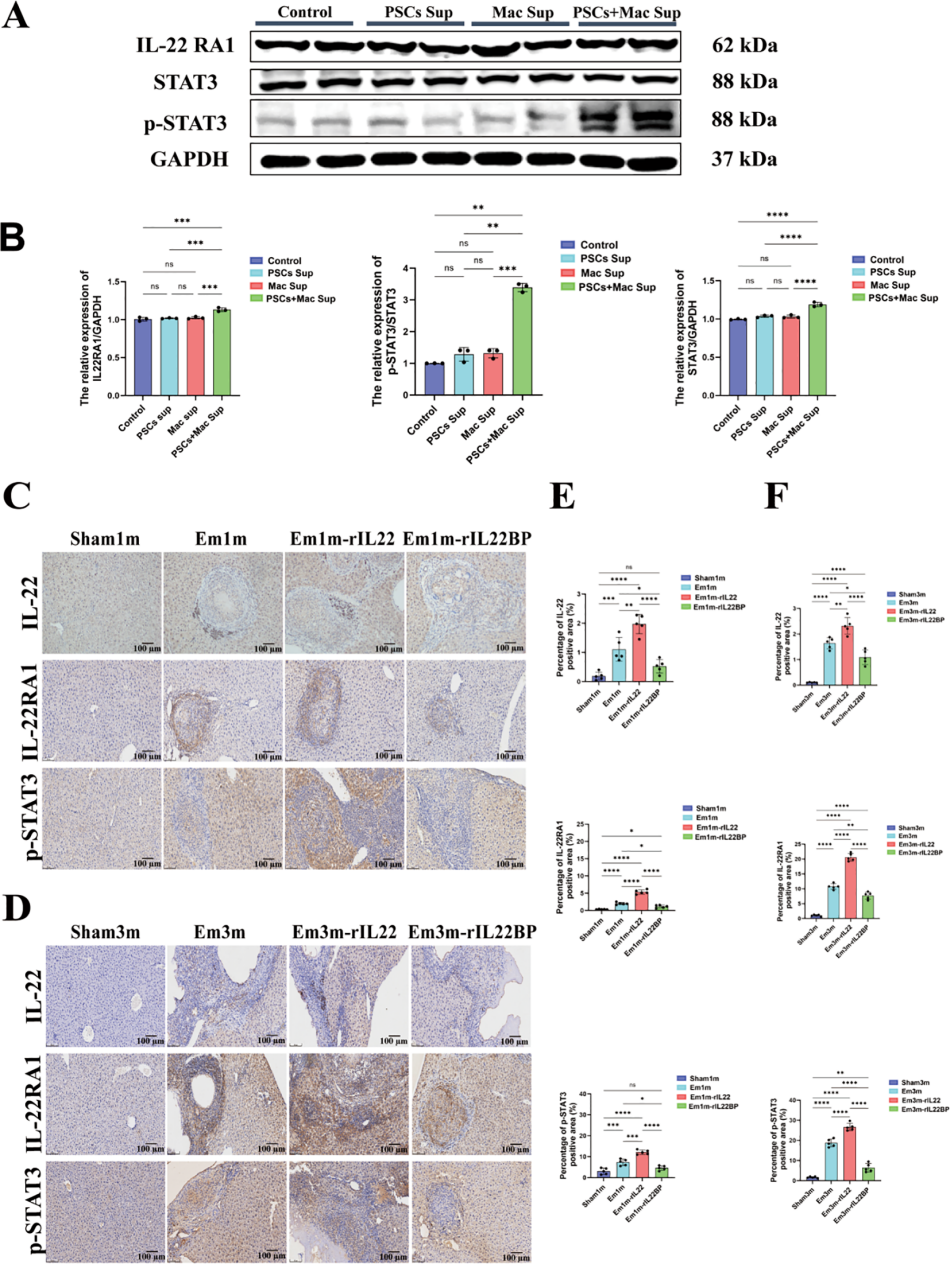
IL-22 promotes CD155 expression through the IL-22RA1-STAT3 signaling axis. **(A)** Representative western blotting images of IL-22RA1, STAT3 and p-STAT3 expression in hepatocytes across experimental groups. **(B)** Statistical results of IL-22RA1, STAT3 and p-STAT3 expression in hepatocytes across experimental groups (n=3). **(C)** Immunohistochemical representative images of IL-22, IL-22RA1 and p-STAT3 in the liver tissues of mice at 1-month post-infection. Scale bar: 100μm. **(D)** Immunohistochemical representative images of IL-22, IL-22RA1 and p-STAT3 in the liver tissues of mice at 3-month post-infection. Scale bar: 100μm. **(E)** Statistical results of the positive area of IL-22, IL-22RA1 and p-STAT3 in the liver of mice at 1-month post-infection (n =5). **(F)** Statistical results of the positive area of IL-22, IL-22RA1 and p-STAT3 in the liver of mice at 3-month post-infection (n =5). *P < 0.05, **P < 0.01, ***P < 0.001, ****P < 0.0001. ns, no significance.

To elucidate the mechanism by which IL-22 regulates CD155 expression in hepatocytes, we examined signaling alterations in AML12 cells after IL-22 stimulation. Previous work has shown that IL-22 up-regulates CD155 via the STAT3 pathway, thereby reducing sorafenib sensitivity in hepatocellular carcinoma cells and impairing NK-cell-mediated tumor cell lysis (25). Consistently, western blotting revealed that PSCs + Mac Sup treatment specifically activated the IL-22-STAT3 axis in AML12 cells, evidenced by significant up-regulation of IL-22RA1 and increased p-STAT3 levels, compared with other groups (**Figures 5A, B**). Similarly, *in vivo* experiments demonstrated that targeted IL-22 intervention modulated STAT3 activity. Compared to the Sham group, the areas positive for IL-22, IL-22RA1, and p-STAT3 were significantly increased in mice of Em1m and Em3m group, with a further elevation observed in the rIL-22 group. Conversely, the rIL-22BP group significantly suppressed the expression of IL-22RA1 and p-STAT3 induced by *E.m* infection (**Figures 5C–F**). Taken together, these data indicate that PSCs-stimulated macrophages secrete IL-22, which triggers STAT3 phosphorylation via IL-22RA1 on hepatocytes. This signaling cascade contributes to the high expression of CD155, thereby fostering an immune-tolerant microenvironment during *E.m* infection.

### Blockade of IL-22 signaling restores CD8^+^T cell function in *E.m*-infected mice

**Figure 6:**
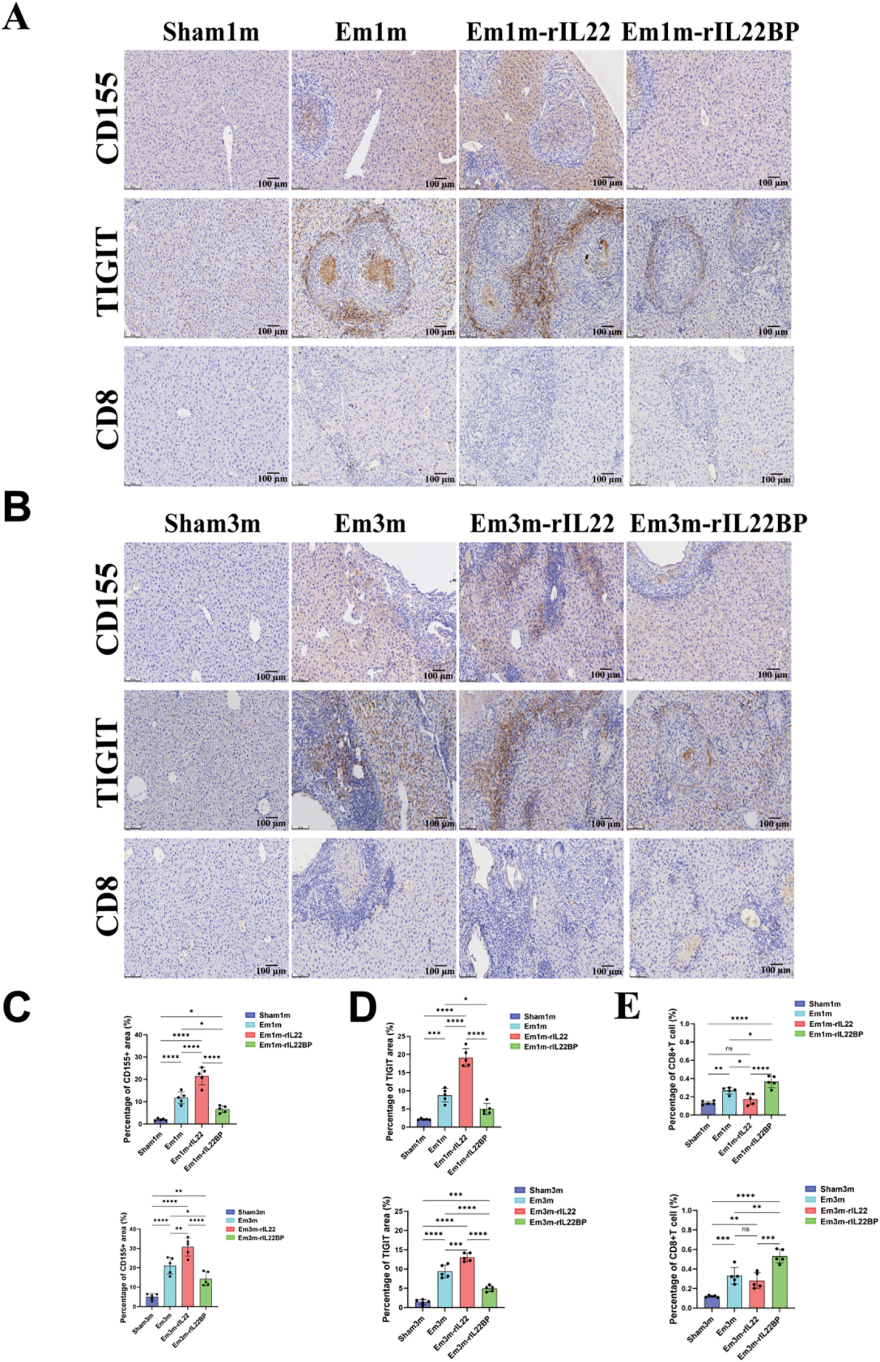
Impact of IL-22 intervention on the immune-exhausted microenvironment in *E.m i*nfected mice. **(A)** Immunohistochemical representative images of CD155, TIGIT and CD8 in the liver tissues of mice at 1-month post-infection. Scale bar: 100μm. **(B)** Immunohistochemical representative images of CD155, TIGIT and CD8 in the liver tissues of mice at 3-month post-infection. Scale bar: 100μm. **(C)** Statistical results of the positive area of CD155 in the liver of mice at 1- and 3-month post-infection (n =5). **(D)** Statistical results of the positive area of TIGIT in the liver of mice at 1- and 3-month post-infection (n =5). **(E)** Statistical results of the positive area of CD8 in the liver of mice at 1- and 3-month post-infection (n =5). **P < 0.01, ***P < 0.001, ****P < 0.0001. ns, no significance.

To elucidate the immunological mechanism by which targeted blockade of IL-22 signaling reverses liver injury in *E.m-*infected mice, we examined changes in key immune molecules after IL-22 inhibition. In both the Em1m and Em3m groups, infection markedly increased IL-22 expression and concurrently up-regulated the immune checkpoint molecules CD155 and its receptor TIGIT, thereby establishing an immune-tolerant microenvironment. Treatment with rIL-22BP significantly suppressed CD155 and TIGIT protein levels (**Figures 6A–D**). Moreover, *E.m* infection reduced CD8^+^ T cell infiltration, and exogenous IL-22 further exacerbated this reduction, indicating that elevated IL-22 impairs CD8^+^ T cell mediated immune killing of parasites. In contrast, rIL-22BP restored CD8^+^ T-cell infiltration (**Figures 6A–B, E**). These findings demonstrate that blockade of IL-22 signaling inhibits CD155/TIGIT mediated T cell exhaustion, reverses CD8^+^ T cell dysfunction, and reshapes the host immune microenvironment to counter *E.m* infection.

## Discussion

AE, a lethal parasitic zoonosis caused by *E.m* infection, poses severe health threats due to its cancer-like infiltrative growth in the liver. Owing to the insidious onset of the disease, the majority of patients are diagnosed at an advanced stage (2). Currently, benzimidazole drugs and surgical resection of the lesion are the main treatment methods for AE. However, these approaches face challenges including significant drug toxicity, postoperative recurrence, and poor patient quality of life (26). Consequently, discovering new therapies is urgent. Although IL-22 modulates immune responses and tissue repair in various diseases, its role in AE progression, particularly in shaping the hepatic immune microenvironment has remained unexplored. Our study reveals that during *E.m* infection, IL-22 induces CD155 expression in hepatocytes, creating an immunosuppressive niche that promotes disease progression. Notably, treatment with rIL-22BP reversed the liver injury in mice. We further identified macrophages as a key cellular source of IL-22, revealing their previously unrecognized role in AE immunopathogenesis. Mechanistically, macrophage-derived IL-22 activates the IL-22RA1/STAT3 axis to upregulate hepatocyte CD155 expression. Disruption of this axis via rIL-22BP blockade inhibits the CD155/TIGIT checkpoint, thereby remodeling the anti-parasite immune microenvironment and enhancing parasite clearance. Collectively, these findings provide compelling evidence supporting immunotherapy as a promising strategy for AE management.

Immune exhaustion is a progressive state of functional impairment in T cells or other immune cells resulting from prolonged antigenic stimulation during chronic infections or cancer (27). It is characterized by decreased effector functions, high expression of inhibitory receptors, metabolic dysregulation, and impaired cytokine production and antibody synthesis (28). Our previous research demonstrated that in AE, an immunosuppressive microenvironment surrounding hepatic lesions facilitates parasite immune evasion and sustains chronic parasitism, with the CD155-TIGIT checkpoint axis mediating T-cell exhaustion (19). However, key microenvironmental drivers activating this pathway remained undefined. Early study in *Echinococcus granulosus* infection suggested IL-22 contributes to host defense by activating Th22 responses and enhancing epithelial barrier function, potentially synergizing with Th17/AhR pathways to regulate immune homeostasis (17). In this study, we observed upregulated IL-22 expression in the peri-lesion tissues and peripheral blood of AE patients and mouse models, positively correlating with CD155 expression and disease severity. This implicates IL-22 as a key molecular link between parasitic infection and CD155-TIGIT-mediated immunosuppression. Critically, we demonstrate that IL-22 drives immune exhaustion and disease progression in AE. Using rIL-22BP to specifically block IL-22 signaling, we achieved significant reductions in parasitic burden in *E.m-*infected mice. Mechanistic investigations revealed that IL-22 blockade attenuated IL-22RA1/STAT3 signaling, downregulated CD155 expression in hepatocytes and enhanced CD8^+^ T cells infiltration. These findings provide direct evidence that IL-22 promotes immune exhaustion via upregulation of CD155, thereby facilitating disease progression. Importantly, our results highlight that targeting the IL-22-IL-22R1 axis effectively restores T cell function and mitigates disease.

IL-22 is a cytokine produced by immune cells that acts on non-immune cells, such as mucosal and skin epithelial cells and hepatocytes (28, 29). Wang et al. found that type 3 innate lymphoid cells (ILC3s) are important producers of IL-22 in the gut, and that they play a key role in maintaining intestinal host-microbiota homeostasis through IL-22 secretion (30). Deng et al. reported that IL-22 derived from T cells and macrophages promotes osteogenic differentiation of human aortic valve mesenchymal stromal cells through activation of JAK3/STAT3 signaling (31). To elucidate the cellular source of IL-22 in *E.m-*infected mice, we depleted macrophages from *E.m*-infected mice, and as macrophage infiltration in the tissues surrounding the lesions decreased, the expression of IL-22 in liver tissues and the level of serum IL-22 were significantly decreased. Further *in vitro* experiments confirmed that co-culturing hepatocytes with supernatants from PSCs-stimulated macrophages (which express high levels of IL-22) significantly upregulated CD155 expression in hepatocytes, consistent with *in vivo* observations. These findings identify macrophages as a major source of IL-22 during *E.m* infection and provide direct evidence that macrophage-derived IL-22 drives CD155 expression.

As a member of the IL-10 family, IL-22 mediates tissue repair and immunosuppression through the downstream IL-22RA1/STAT3 signaling axis (25). As a key mediator for IL-22 signal transduction, sustained activation of STAT3 may drive the pro-inflammatory or carcinogenic effects of IL-22 (32). In our previous work, we found that the CD155-TIGIT axis induced immune depletion in AE through both direct inhibition of TCR signaling and competitive blockade of the co-stimulatory receptor CD226 (20). Building on this, the present study further identified the activation of the IL-22-STAT3 signaling pathway as a central component driving CD155 overexpression in hepatocytes, which in turn induces T-cell exhaustion via the CD155-TIGIT axis. In chronic liver disease, IL-22 has a dual role, exhibiting both protective functions and potentially participating in pathogenic processes. On the one hand, IL-22 attenuates liver fibrosis by activating the Nrf2-ARE pathway, reducing the level of oxidative stress and inhibiting the activation and proliferation of hepatic stellate cells (HSC) (33). In non-alcoholic fatty liver disease (NAFLD) or metabolic dysfunction-associated steatohepatopathy (MASLD), IL-22 exerts protective effects by regulating lipid metabolism and suppressing inflammatory responses (34). On the other hand, in the HCC microenvironment, IL-22 is mainly secreted by CD4^+^ T cells and accelerates tumor progression by promoting angiogenesis and immune escape (35). Similar, in our study, IL-22 also acted as a key activator, inducing an immune-exhausted microenvironment in *E.m* infection and promoting immune escape of the parasite.

Although this study suggests that IL-22 is involved in the establishment of an immune exhaustion microenvironment in *E.m* infection by modulating CD155 expression in hepatocytes, many questions remain unanswered and warrant further investigation. First, our study preliminarily demonstrated that IL-22 is primarily derived from macrophages. However, whether other cell types also contribute to IL-22 secretion in *E.m* infection needs to be further demonstrated. As previously mentioned, IL-22 can be produced by various cell types depending on the disease context, and our study does not exclude the possibility that other cells may also be a source of IL-22. Future studies using advanced techniques based on single-cell analysis such as single-cell transcriptome sequencing could help to clarify this issue. Secondly, as an important immunomodulatory molecule, IL-22 typically exhibits pleiotropic effects. The present study clarified only one of the mechanisms by which it has the ability to regulate CD155 expression in hepatocytes. However, IL-22 is a pleiotropic cytokine with complex and diverse roles in immunomodulation, tissue repair, and inflammatory responses (30, 35–37). Whether IL-22 exerts additional biological functions during *E.m* infection remains to be elucidated in further studies. Finally, this study preliminarily demonstrated that rIL-22BP has the therapeutic potential in attenuating *E.m* infection. However, the optimal timing of intervention, dosage, and potential toxicities of rIL-22BP require further investigation before clinical application.

In conclusion, the present study reveals that macrophage-derived IL-22 induces CD155 expression in hepatocytes through activation of the STAT3 pathway, thereby promoting the establishment of an immune exhausted microenvironment during *E.m* infection, which in turn facilitates long-term parasitism. Blocking the immunosuppressive activity of IL-22 by IL-22BP can effectively remodel the tissue immune microenvironment and promotes parasite clearance by host immune cells.

## Data Availability

The original contributions presented in the study are included in the article/**Supplementary Material**. Further inquiries can be directed to the corresponding authors.

## References

[1] Budke CM Casulli A Kern P Vuitton DA . Cystic and alveolar echinococcosis: Successes and continuing challenges. PloS Negl Trop Dis. (2017) 11:e0005477. doi: 10.1371/journal.pntd.0005477, PMID: 28426657 PMC5398475

[2] Wen H Vuitton L Tuxun T Li J Vuitton DA Zhang W . Echinococcosis: advances in the 21st century. Clin Microbiol Rev. (2019) 32:e00075-18. doi: 10.1128/CMR.00075-18, PMID: 30760475 PMC6431127

[3] Wang H Zhang CS Fang BB Hou J Li WD Li ZD . Dual role of hepatic macrophages in the establishment of the echinococcus multilocularis metacestode in mice. Front Immunol. (2020) 11:600635. doi: 10.3389/fimmu.2020.600635, PMID: 33488594 PMC7820908

[4] Godot V Harraga S Podoprigora G Liance M Bardonnet K Vuitton DA . IFN alpha-2a protects mice against a helminth infection of the liver and modulates immune responses. Gastroenterology. (2003) 124:1441–50. doi: 10.1016/s0016-5085(03)00273-7, PMID: 12730883

[5] Wu Y Min J Ge C Shu J Tian D Yuan Y . Interleukin 22 in liver injury, inflammation and cancer. Int J Biol Sci. (2020) 16:2405–13. doi: 10.7150/ijbs.38925, PMID: 32760208 PMC7378634

[6] Wan R Srikaram P Xie S Chen Q Hu C Wan M . PPARγ attenuates cellular senescence of alveolar macrophages in asthma-COPD overlap. Respir Res. (2024) 25:174. doi: 10.1186/s12931-024-02790-6, PMID: 38643159 PMC11032609

[7] Liu Y Verma VK Malhi H Gores GJ Kamath PS Sanyal A . Lipopolysaccharide downregulates macrophage-derived IL-22 to modulate alcohol-induced hepatocyte cell death. Am J Physiol Cell Physiol. (2017) 313:C305–C13. doi: 10.1152/ajpcell.00005.2017, PMID: 28637673 PMC5625090

[8] Giannou AD Kempski J Shiri AM Lucke J Zhang T Zhao L . Tissue resident iNKT17 cells facilitate cancer cell extravasation in liver metastasis via interleukin-22. Immunity. (2023) 56:125–42 e12. doi: 10.1016/j.immuni.2022.12.014, PMID: 36630911 PMC9839362

[9] Qin J Zhu W Zhou W . Navigating the paradox of IL-22: friend or foe in hepatic health? J Gastroenterol Hepatol. (2025) 40:1393–408. doi: 10.1111/jgh.16991, PMID: 40358483

[10] Abdelnabi MN Flores Molina M Soucy G Quoc-Huy Trinh V Bédard N Mazouz S . Sex-dependent hepatoprotective role of IL-22 receptor signaling in non-alcoholic fatty liver disease-related fibrosis. Cell Mol Gastroenterol Hepatol. (2022) 14:1269–94. doi: 10.1016/j.jcmgh.2022.08.001, PMID: 35970323 PMC9596743

[11] Khawar MB Azam F Sheikh N Abdul Mujeeb K . How does interleukin-22 mediate liver regeneration and prevent injury and fibrosis? J Immunol Res. (2016) 2016:2148129. doi: 10.1155/2016/2148129, PMID: 28050571 PMC5168458

[12] Kotenko SV Izotova LS Mirochnitchenko OV Esterova E Dickensheets H Donnelly RP . Identification, cloning, and characterization of a novel soluble receptor that binds IL-22 and neutralizes its activity. J Immunol. (2001) 166:7096–103. doi: 10.4049/jimmunol.166.12.7096, PMID: 11390454

[13] Huber S Gagliani N Zenewicz LA Huber FJ Bosurgi L Hu B . IL-22BP is regulated by the inflammasome and modulates tumorigenesis in the intestine. Nature. (2012) 491:259–63. doi: 10.1038/nature11535, PMID: 23075849 PMC3493690

[14] Lücke J Sabihi M Zhang T Bauditz LF Shiri AM Giannou AD . The good and the bad about separation anxiety: roles of IL-22 and IL-22BP in liver pathologies. Semin Immunopathol. (2021) 43:591–607. doi: 10.1007/s00281-021-00854-z, PMID: 33851257 PMC8443499

[15] Su S Qin S Xian X Huang F Huang Q Zhang D . Interleukin-22 regulating Kupffer cell polarization through STAT3/Erk/Akt crosstalk pathways to extenuate liver fibrosis. Life Sci. (2021) 264:118677. doi: 10.1016/j.lfs.2020.118677, PMID: 33129875

[16] Jiang Z Li W Yu S Wang X Jiang H Bai C . IL-22 relieves hepatic ischemia-reperfusion injury by inhibiting mitochondrial apoptosis based on the activation of STAT3. Int J Biochem Cell Biol. (2024) 166:106503. doi: 10.1016/j.biocel.2023.106503, PMID: 38036287

[17] Li LL Jiang HQ Fang HR Dong D Chen C Hou J . Early infection of Echinococcus granulosus promotes IL-22 production in mice. Immunol J. (2019) 35:315-20. doi: 10.13431/j.cnki.immunol.j.20190049

[18] Briukhovetska D Suarez-Gosalvez J Voigt C Markota A Giannou AD Schubel M . T cell-derived interleukin-22 drives the expression of CD155 by cancer cells to suppress NK cell function and promote metastasis. Immunity. (2023) 56:143–61 e11. doi: 10.1016/j.immuni.2022.12.010, PMID: 36630913 PMC9839367

[19] Zhang C Lin R Li Z Yang S Bi X Wang H . Immune exhaustion of T cells in alveolar echinococcosis patients and its reversal by blocking checkpoint receptor TIGIT in a murine model. Hepatology. (2020) 71:1297–315. doi: 10.1002/hep.30896, PMID: 31410870

[20] Zhang C Wang H Li J Hou X Li L Wang W . Involvement of TIGIT in natural killer cell exhaustion and immune escape in patients and mouse model with liver echinococcus multilocularis infection. Hepatology. (2021) 74:3376–93. doi: 10.1002/hep.32035, PMID: 34192365

[21] Kern P Wen H Sato N Vuitton DA Gruener B Shao Y . WHO classification of alveolar echinococcosis: principles and application. Parasitol Int. (2006) 55 Suppl:S283–7. doi: 10.1016/j.parint.2005.11.041, PMID: 16343985

[22] Mulati M Yang N Xue J Li L Zhang X Liu H . ADSCs attenuate Liver fibrosis via inducing HSC senescence: validation in dual-etiology models. PloS Negl Trop Dis. (2025) 19:e0013094. doi: 10.1371/journal.pntd.0013094, PMID: 40402971 PMC12148229

[23] Xiang X Feng D Hwang S Ren T Wang X Trojnar E . Interleukin-22 ameliorates acute-on-chronic liver failure by reprogramming impaired regeneration pathways in mice. J Hepatol. (2020) 72:736–45. doi: 10.1016/j.jhep.2019.11.013, PMID: 31786256 PMC7085428

[24] Wei P Zhang Y Zhang T Yang Z Hou J Tian M . Research on the expression changes and inhibitory effects of CD47 and SIRPα in macrophages infected with Echinococcus multilocularis. Chin J Parasitol Parasitic Diseases. (2025) 43:84–90. doi: 10.12140/j.issn.1000-7423.2025.01.013

[25] Chen J Sun S Li H Cai X Wan C . IL-22 signaling promotes sorafenib resistance in hepatocellular carcinoma via STAT3/CD155 signaling axis. Front Immunol. (2024) 15:1373321. doi: 10.3389/fimmu.2024.1373321, PMID: 38596684 PMC11003268

[26] Xu K Ahan A . A new dawn in the late stage of alveolar echinococcosis “parasite cancer. Med Hypotheses. (2020) 142:109735. doi: 10.1016/j.mehy.2020.109735, PMID: 32344283

[27] Bonté PE Metoikidou C Heurtebise-Chretien S Arribas YA Sutra Del Galy A Ye M . Selective control of transposable element expression during T cell exhaustion and anti-PD-1 treatment. Sci Immunol. (2023) 8:eadf8838. doi: 10.1126/sciimmunol.adf8838, PMID: 37889984

[28] Saeidi A Zandi K Cheok YY Saeidi H Wong WF Lee CYQ . T-cell exhaustion in chronic infections: reversing the state of exhaustion and reinvigorating optimal protective immune responses. Front Immunol. (2018) 9:2569. doi: 10.3389/fimmu.2018.02569, PMID: 30473697 PMC6237934

[29] Tang KY Lickliter J Huang ZH Xian ZS Chen HY Huang C . Safety, pharmacokinetics, and biomarkers of F-652, a recombinant human interleukin-22 dimer, in healthy subjects. Cell Mol Immunol. (2019) 16:473–82. doi: 10.1038/s41423-018-0029-8, PMID: 29670279 PMC6474205

[30] Wang Y Ngo VL Zou J Gewirtz AT . Commensal bacterial outer membrane protein A induces interleukin-22 production. Cell Rep. (2024) 43:114292. doi: 10.1016/j.celrep.2024.114292, PMID: 38823020 PMC11247541

[31] Deng H Li H Liu Z Shen N Dong N Deng C . Pro-osteogenic role of interleukin-22 in calcific aortic valve disease. Atherosclerosis. (2024) 388:117424. doi: 10.1016/j.atherosclerosis.2023.117424, PMID: 38104486

[32] Cineus R Luo Y Saliutina M Manna S Cancino CA Velasco Blázquez L . The IL-22-oncostatin M axis promotes intestinal inflammation and tumorigenesis. Nat Immunol. (2025) 26:837–53. doi: 10.1038/s41590-025-02149-z, PMID: 40447860 PMC12133592

[33] Xu X Zhao H Zhang J Yan H Liu X Huo J . Interleukin-22 ameliorates alcohol-associated liver fibrosis via Nrf2-ARE signaling: mechanistic insights and clinical correlations. Clin Res Hepatol Gastroenterol. (2025) 49:102617. doi: 10.1016/j.clinre.2025.102617, PMID: 40449584

[34] Zhang P Liu J Lee A Tsaur I Ohira M Duong V . IL-22 resolves MASLD via enterocyte STAT3 restoration of diet-perturbed intestinal homeostasis. Cell Metab. (2024) 36:2341–54 e6. doi: 10.1016/j.cmet.2024.08.012, PMID: 39317186 PMC11631175

[35] Giannou AD Lücke J Kleinschmidt D Shiri AM Steglich B Nawrocki M . A critical role of the IL-22-IL-22 binding protein axis in hepatocellular carcinoma. Cancers (Basel). (2022) 14:6019. doi: 10.3390/cancers14246019, PMID: 36551508 PMC9775560

[36] Zhao N Liu C Li N Zhou S Guo Y Yang S . Role of Interleukin-22 in ulcerative colitis. BioMed Pharmacother. (2023) 159:114273. 36696801 10.1016/j.biopha.2023.114273

[37] Scopelliti F Cattani C Gimmelli R Dimartino V Lalli C Papoff G . Profiling of human IL-22+ T cell clones from patients affected with Schistosoma mansoni: Insights into macrophage regulation and liver fibrosis. PloS Negl Trop Dis. (2025) 19:e0013132. doi: 10.1371/journal.pntd.0013132, PMID: 40446079 PMC12176296

