# Supplementary material for: IL-22-STAT3-CD155 axis in alveolar echinococcosis: a pivotal role in immune exhaustion and therapeutic potential
Source: Front Immunol. 2026 Jan 5;16:1674904. doi: 10.3389/fimmu.2025.1674904 (PMC12813123; doi:10.3389/fimmu.2025.1674904)

Supplementary Material

Supplementary Table 1. Baseline clinical characteristics of AE patients studied

Patient No.	Age (years)	Gender (F/M)	WHO PNM stage	ALP (U/L)	ALT (U/L)	AST (U/L)	Lesion's Location and Diameter (cm)	Biliary Complication	Invasion of Neighboring Organs and Metastases	Prior Benzimidazole Use	Sample type
**1**	52	F	P4N0M0	97.0	71.0	67.0	RL, 6.4×6.0; 6.0×3.1	None	None	Albendazole	Serum, CLT, DLT
**2**	65	M	P2N1M0	365.8	198.0	276.0	RL, 12.0×5.8	obstructive jaundice	Kidney	Albendazole	Serum
**3**	44	F	P2N0M0	50.0	228	433.0	LL+RL, 8.2×8.0	None	None	None	Serum, CLT
**4**	48	M	P4N1M0	87.0	56.2	79.2	RL, 7.8×5.7	None	adrenal gland	None	Serum, CLT, DLT
**5**	46	F	P3N1M0	331.1	215.6	100.1	RL, 10.7×9.7	None	mediastinum	None	Serum
**6**	32	M	P2N0M0	86.8	33.6	30.7	LL, 7.8×4.7	obstructive jaundice	None	None	Serum, CLT
**7**	47	F	P4N0M0	97.1	18.1	19.1	LL+RL, 9.9×3.9	None	None	None	Serum, CLT, DLT
**8**	52	F	P4N0M0	151.0	28.1	30.8	LL, 4.0×3.9, 4.3×3.2, 4.8×3.9	None	None	Albendazole	Serum
**9**	44	F	P3N1MO	38.0	7.2	10.5	LL, 4.8×4.4; 5.0×5.3	None	pancreas	None	Serum
**10**	45	M	P2N0M0	542.4	354.5	123.7	RL, 12.3×11.9	None	None	None	Serum, CLT, DLT
**11**	33	F	P3N0M0	121.0	101.0	62.0	LL, 4.9×3.7	None	None	Albendazole	Serum, CLT, DLT

**12**	26	M	P4N0M0	267.3	65.5	45.0	LL+RL, 19.7×14.2	None	None	None	Serum
**13**	21	F	P2N0M0	120.1	12.9	18.5	LL, 2.4×2.7; 11.1×9.4	None	None	None	Serum, CLT, DLT
**14**	23	M	P4N1M0	409.8	151.0	84.0	RL, 12.4×14.3; 8.1×8.5	None	pancreas	None	Serum, CLT
**15**	44	F	P4N0M0	861.8	152.1	138.8	RL, 8.9×10.4	None	None	None	Serum, CLT, DLT
**16**	41	M	P3N0M0	72.0	22.0	23.0	RL, 10.3×7.4	None	None	None	Serum, CLT
**17**	60	F	P2N0M0	126.0	277.0	363.0	RL, 15.0×9.3	None	None	None	Serum
**18**	29	F	P2N1M1	132.0	92.0	61.0	LL+RL, 14.0×14.0	None	kidney, lung	Albendazole	Serum, CLT, DLT
**19**	49	M	P3N1M0	243.1	49.7	41.7	LL+RL, 8.4×8.1; 15.4×10.6	None	Kidney	Albendazole	Serum, CLT, DLT
**20**	66	F	P3N0M0	111.2	11.1	13.5	RL, 17.4×6.0	None	None	None	Serum, CLT
**21**	32	F	P3N0M0	109.0	22.9	25.2	LL, 18.2×12.6	None	None	Albendazole	Serum, CLT, DLT
**22**	36	M	P2N0M0	108.0	21.0	14.5	RL, 7.9×7.6	None	None	None	Serum, CLT, DLT
**23**	15	M	P4N0M0	207.0	54.0	51.0	RL, 11.7×9.7	obstructive jaundice	None	None	Serum, CLT
**24**	33	F	P3N1M0	339.1	45.7	69.7	RL, 20.0×20.0	obstructive jaundice	adrenal gland	None	Serum
**25**	19	M	P2N1M0	171.2	12.0	27.5	RL, 10.2×8.1	None	Kidney	Albendazole	Serum, CLT, DLT
**26**	22	F	P3N0M0	222.6	56.1	44.0	LL, 11.8×8.1	None	None	None	Serum, CLT, DLT
**27**	57	M	P3N0M0	41.9	18.4	17.6	RL, 4.0×3.0	None	None	None	Serum, CLT, DLT

**28**	34	F	P4N1M1	49.8	27.1	25.2	RL, 20.0×21.0	None	mediastinum, lung	None	Serum, CLT
**29**	61	M	P2N0M0	1449.8	116.1	149.1	RL, 7.7×7.7	None	None	None	Serum, CLT
**30**	26	F	P4N0M0	95.2	65.7	50.2	RL, 12.0×7.0	obstructive jaundice	None	None	Serum, CLT, DLT
**31**	51	F	P2N0M1	858.0	89.6	114.6	LL, 6.6×5.2; 5.8×4.6	None	vertebra	None	Serum, CLT
**32**	18	M	P3N1M1	312.9	26.9	32.4	LL, 8.8×7.0	None	mediastinum, lung	None	Serum, CLT, DLT
**33**	33	M	P2N1M0	98.8	55.9	50.9	RL, 11.0×7.0	None	pancreas	Albendazole	Serum, CLT
**34**	57	M	P4N0M1	99.2	13.0	23.4	LL, 5.9×5.4; 10.1×7.4	None	Lung	None	Serum, CLT, DLT
**35**	22	M	P3N1M0	237.1	18.4	20.7	RL, 12.2×7.4	None	adrenal gland, lung	None	Serum, CLT, DLT
**36**	24	F	P4N1M0	75.5	84.6	117.0	LL+RL, 5.5×6.2	None	mediastinum	Albendazole	Serum, CLT, DLT
**37**	35	M	P4N1M0	149.4	28.7	22.3	LL, 3.8×3.0; 12.6×5.3	None	peritoneal cavity	Albendazole	Serum, CLT
**38**	31	F	P4N0M0	253.8	39.8	44.4	LL+RL, 15.8×12.2	obstructive jaundice	None	None	Serum, CLT, DLT
**39**	26	M	P4N1M1	331.7	17.4	18.5	RL, 8.5×9.5	None	peritoneal cavity, lung	None	Serum, CLT, DLT
**40**	38	F	P4N1M0	86.0	10.5	21.6	RL, 10.5×10.6; 0.59×0.79	None	Kidney	Albendazole	Serum
**41**	60	F	P4N0M0	177.2	23.5	27.2	RL, 13.2×11.4; 3.6×3.2	None	None	None	Serum
**42**	36	M	P4N1M0	77.9	29.4	20.4	RL, 14.3×12.0	None	peritoneal cavity	None	Serum, CLT, DLT
**43**	30	F	P4N0M0	180.8	17.8	25.9	RL, 17.2×15.0; 3.7×2.3	None	None	Albendazole	Serum
**44**	51	M	P2N0M0	49.0	55.0	45.0	LL, 11.0×10.7	None	None	None	Serum, CLT, DLT

**45**	48	F	P4N0M0	151.6	51.7	50.0	LL, 13.1×10.6	None	None	Albendazole	Serum
**46**	63	M	P3N0M0	59	47.2	39.3	LL+RL, 10.9×9.0	None	None	None	Serum
**47**	51	M	P4N1M0	62.9	19.7	23.4	RL, 7.0×6.0; 2.0×1.0	None	mediastinum	None	Serum, CLT, DLT
**48**	45	M	P4N0M0	253.7	83.1	182.0	RL, 20.5×14.7	None	None	Albendazole	Serum, CLT, DLT
**49**	37	F	P1N0M0	44.9	24.7	21.1	RL, 9.1×10.4	None	None	Albendazole	Serum, CLT
**50**	28	M	P3N0M0	215.8	26.0	24.6	LL+RL, 12.8×10.8	None	None	None	Serum
**51**	57	M	P2N0M0	62.8	39.3	21.1	RL, 3.0×3.0	None	None	None	Serum, CLT, DLT
**52**	24	M	P2N0M0	153.6	32.1	24.4	RL, 12.0×12.0	None	None	None	Serum
**53**	22	M	P3N0M0	608.3	134.4	109.1	LL+RL, 10.0×10.0	None	None	None	Serum
**54**	23	F	P3N0M0	319.2	62.3	63.8	RL, 11.3×7.6	obstructive jaundice	None	None	Serum, CLT

Abbreviations: F, female; M, male; P = location of the parasitic mass in the liver, N = involvement of neighboring organs, M = metastases; ALP, alkaline phosphatase; ALT, alanine aminotransferase; AST, aspartate aminotransferase; LL, left liver lobe; RL, right liver lobe; CLT, close liver tissue; DLT, distance liver tissue.

Supplementary Figure 1. The influence of IL-22 intervention on body and liver weight in *E.m* infected mice.

(A) Statistical results of the body weight, liver weight and the ratio of liver/body weight in mice at 1-month post-infection (n =5). (B) Statistical results of the body weight, liver weight and the ratio of liver/body weight in mice at 3-month post-infection (n =5). **P* < 0.05, ***P* < 0.01, ****P* < 0.001, *****P* < 0.0001. ns, no significance.

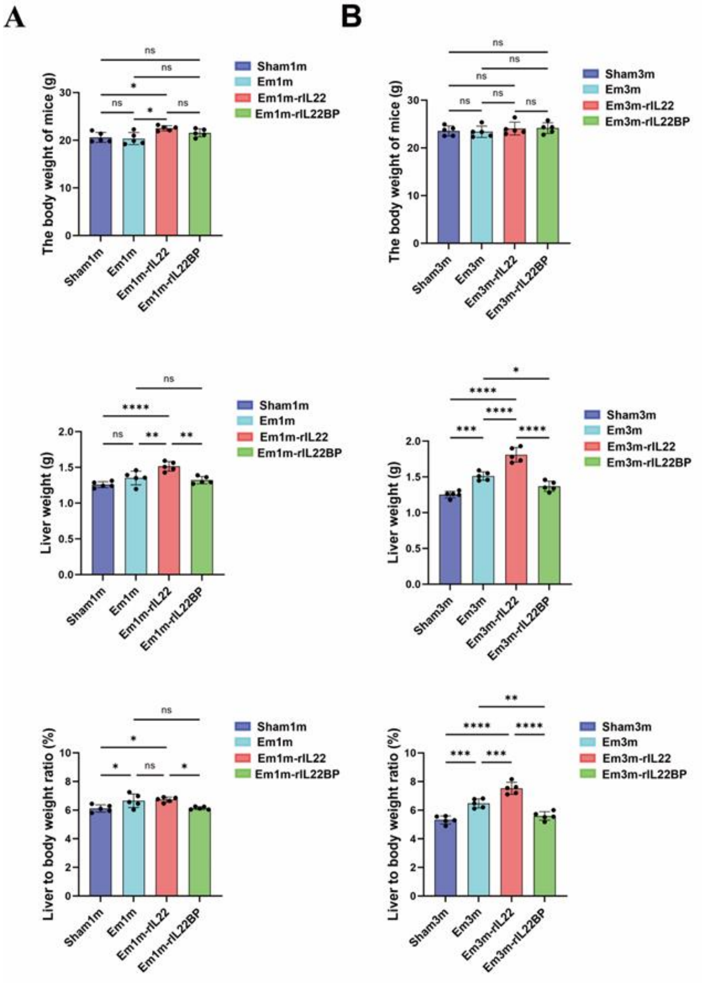

Supplement: Supplementary file 1 [file DataSheet1.pdf]
