# Supplementary figures and images for: IL-22-STAT3-CD155 axis in alveolar echinococcosis: a pivotal role in immune exhaustion and therapeutic potential
Source: Front Immunol. 2026 Jan 5;16:1674904. doi: 10.3389/fimmu.2025.1674904 (PMC12813123; doi:10.3389/fimmu.2025.1674904)

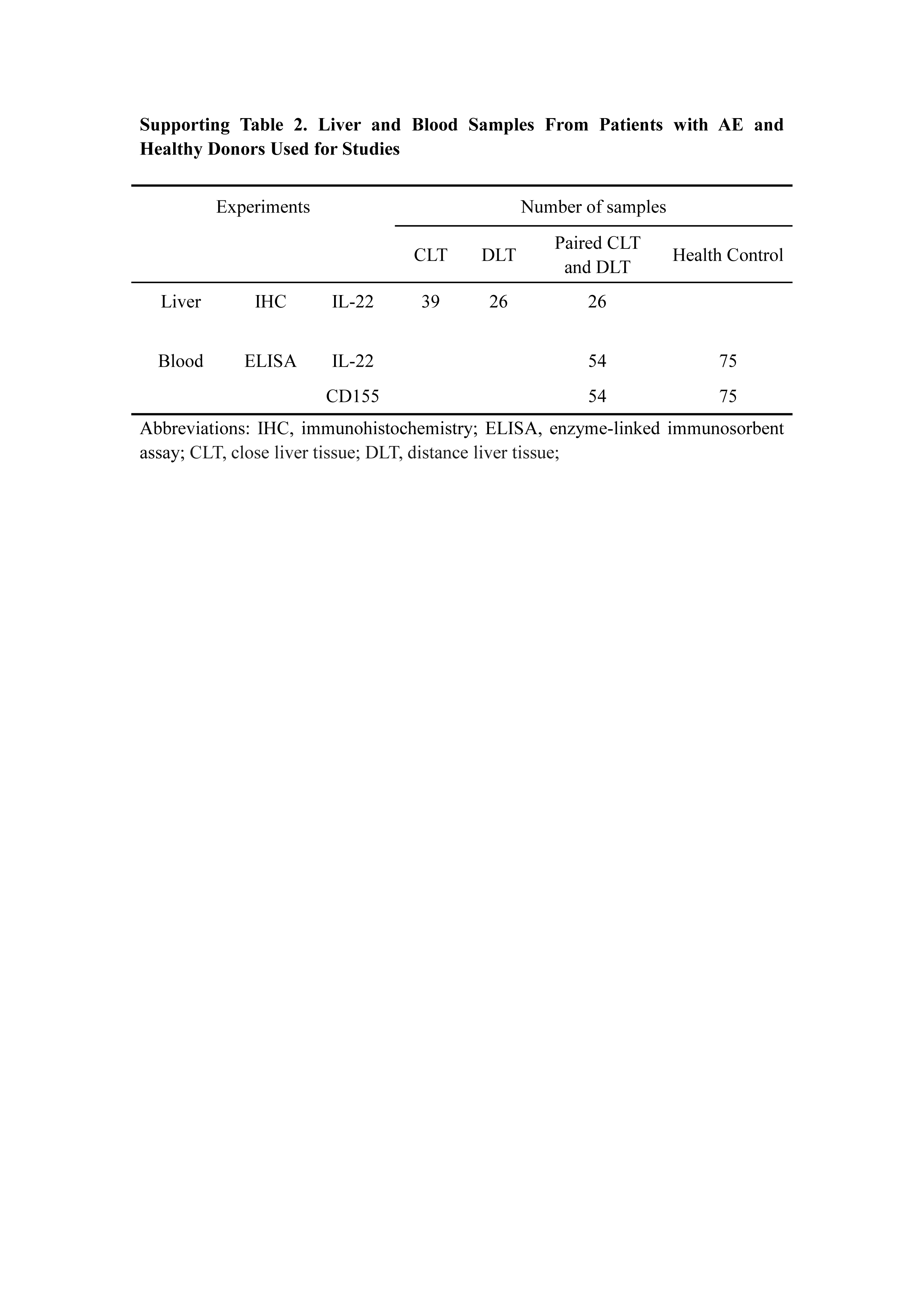

Supplement: Supplementary file 2 [file Supplementaryfile1.tif]
